# A handy approximate solution for a squeezing flow between two infinite plates by using of Laplace transform-homotopy perturbation method

**DOI:** 10.1186/2193-1801-3-421

**Published:** 2014-08-10

**Authors:** Uriel Filobello-Nino, Hector Vazquez-Leal, Juan Cervantes-Perez, Brahim Benhammouda, Agustin Perez-Sesma, Luis Hernandez-Martinez, Victor Manuel Jimenez-Fernandez, Agustin Leobardo Herrera-May, Domitilo Pereyra-Diaz, Antonio Marin-Hernandez, Jesus Huerta Chua

**Affiliations:** Electronic Instrumentation and Atmospheric Sciences School, Universidad Veracruzana, Circuito Gonzalo Aguirre Beltrán S/N, 9100 Xalapa, Veracruz Mexico; Higher Colleges of Technology, Abu Dhabi Men’s College, P.O. Box 25035, Abu Dhabi, United Arab Emirates; National Institute for Astrophysics, Optics and Electronics, Luis Enrique Erro #1, Sta. María Tonantzintla, 72840 Puebla, Mexico; Micro and Nanotechnology Research Center, Universidad Veracruzana, Calzada Ruiz Cortines 455, 94292 Boca del Rio, Veracruz Mexico; Department of Artificial Intelligence, Universidad Veracruzana, Sebastián Camacho No. 5 Centro, 91000 Xalapa, Veracruz Mexico; Civil Engineering School, Universidad Veracruzana, Venustiano Carranza S/N, Col. Revolucion, 93390 Poza Rica, Veracruz Mexico

**Keywords:** Laplace transform homotopy perturbation method, Nonlinear fluid problems, Power series

## Abstract

This article proposes Laplace Transform Homotopy Perturbation Method (LT-HPM) to find an approximate solution for the problem of an axisymmetric Newtonian fluid squeezed between two large parallel plates. After comparing figures between approximate and exact solutions, we will see that the proposed solutions besides of handy, are highly accurate and therefore LT-HPM is extremely efficient.

## Introduction

Although the studies of squeezing flows have their origins in the 19 th century, at present time, it is an issue of considerable importance due to its practical applications in different areas such as physical, biophysical, chemical engineering, and food industry, also they are relevant in liquid metal lubrication theory, polymer processing, compression and injection molding, among many others.

The goal of this study is to find an approximate solution for the problem of squeezing flow between two infinite parallel plates slowly approaching each other. As mentioned in (Ran et al. 2009) these fluids are of paramount importance, in hydrodynamic lubrication theory. Thus, (Langlois 1962) and (Salbu 1964) analyzed isothermal compressible squeeze films neglecting inertial effects, while (Thorpe 1967), found an explicit solution, taking into account these effects. Also have been found some numerical solutions to these problems, such as those provided in (Verma 1981) and (Singh et al. 1990). Additionally, (Rajagopal & Gupta 1981) and (Dandapat & Gupta 1991) extended the previous investigations for the case of flow between rotating parallel plates.

Laplace Transform (L.T.) (or operational calculus) has played an important role in mathematics, not only for its theoretical interest, but also because its methods let to solve, in a simpler fashion, many problems in science and engineering, in comparison with other mathematical techniques (Spiegel 1988). In particular the Laplace Transform is useful for solving linear ordinary differential equations with constant coefficients, and initial conditions, but also can be used to solve some cases of differential equations with variable coefficients and partial differential equations (Spiegel 1988). On the other hand, applications of L.T. for nonlinear ordinary differential equations mainly focus to find approximate solutions, thus in reference (Aminikhan & Hemmatnezhad 2012) was reported a combination of Homotopy Perturbation (HPM) and L.T. methods (LT-HPM), in order to obtain highly accurate solutions for these equations. However, just as with L.T; LT-HPM method has been used mainly to find solutions to problems with initial conditions (Aminikhan & Hemmatnezhad 2012; Aminikhah 2012), because it is directly related with them. Therefore this paper presents the application of LT-HPM, in the search for an approximate solution of the higher order nonlinear ordinary differential equation, which describes a squeezing flow between two infinite plates with, mixed boundary conditions defined on a finite interval (Filobello-Nino et al. 2013).

The case of equations with boundary conditions on infinite intervals, has been studied in some articles, and correspond often to problems defined on semi-infinite ranges (Hossein 2011; Khan et al. 2011). However the methods of solving these problems, are different from what will be presented in this paper (Filobello-Nino et al. 2013). The importance of research on nonlinear differential equations is that many phenomena, practical or theoretical, are of nonlinear nature. In recent years, several methods focused to find approximate solutions to nonlinear differential equations, as an alternative to classical methods, have been reported, such those based on: variational approaches (Assas 2007; He 2007; Kazemnia et al. 2008; Noorzad et al. 2008), tanh method (Evans & Raslan 2005), exp-function (Xu 2007; Mahmoudi et al. 2008), Adomian’s decomposition method (Adomian 1988; Babolian & Biazar 2002; Kooch & Abadyan 2012; Kooch & Abadyan 2011; Vanani et al. 2011; Chowdhury 2011), parameter expansion (Zhang & Xu 2007), homotopy perturbation method (Vazquez-Leal 2014; Marinca & Herisanu 2011; He 1998; He 1999; He 2006a; He 2008; Belendez et al. 2009; He 2000; El-Shaed 2005; He 2006b; Ganji et al. 2009; Ganji et al. 2008; Fereidon et al. 2010; Sharma & Methi 2011; Hossein 2011; Vazquez-Leal et al. 2012a; Vazquez-Leal et al. 2012b; Filobello-Niño et al. 2012a; Biazar & Aminikhan 2009; Biazar & Ghazvini 2009; Filobello-Niño et al. 2012b; Khan & Wu 2011; Madani et al. 2011; Aminikhan & Hemmatnezhad 2012; Aminikhah 2012; Khan et al. 2011; Filobello-Nino et al. 2013), homotopy analysis method (Patel et al. 2012), and perturbation method (Filobello-Niño et al. 2013a) among many others. Also, a few exact solutions to nonlinear differential equations have been reported occasionally (Filobello-Niño et al. 2013b).

The paper is organized as follows. In Standard HPM, we introduce the basic idea of standard HPM method. For Basic Idea of Laplace Transform Homotopy Perturbation Method (LT-HPM) we introduce Laplace transform homotopy perturbation method. Additionally in Governing equations the basic equations for the flow in study are derived. Case Study present the application of LT-HPM method, in the search for an approximate solution for the higher order nonlinear ordinary differential equation, which describes a squeezing flow between two infinite plates. Besides a discussion on the results is presented in Discussion. Finally, a brief conclusion is given in Conclusions.

## Standard HPM

The standard homotopy perturbation method (HPM) was proposed by Ji Huan He, it was introduced like a powerful tool to approach various kinds of nonlinear problems. The Homotopy Perturbation Method (HPM) is considered as a combination of the classical perturbation technique and the homotopy (whose origin is in the topology), but not restricted to small parameters as occur with traditional perturbation methods. For example, HPM method requires neither small parameter nor linearization, but only few iterations to obtain highly accurate solutions (He 1998; He 1999).

To figure out how HPM works, consider a general nonlinear differential equation in the form
1

with the following boundary conditions
2

where *A* is a general differential operator, *B* is a boundary operator, *f*(*r*) a known analytical function and Γ is the domain boundary for Ω. *A* can be divided into two operators *L* and *N*, where *L* is linear and *N* nonlinear; so that (1) can be rewritten as
3

Generally, a homotopy can be constructed as (He 1998; He 1999)
4

or
5

where p is a homotopy parameter, whose values are within range of 0 and 1, *u*_0_ is the first approximation for the solution of (3) that satisfies the boundary conditions.

Assuming that solution for (4) or (5) can be written as a power series of *p* as
6

Substituting (6) into (5) and equating identical powers of *p* terms, there can be found values for the sequence *v*_0_, *v*_1_, *v*_2_,…

When *p* → 1, it yields the approximate solution for (1) in the form
7

## Basic idea of Laplace Transform Homotopy Perturbation Method (LT-HPM)

The objective of this section is to show, how LT-HPM, can be employed to find analytical approximate solutions of Ordinary Differential Equations (ODE, s), as (3).

For this purpose LT-HPM follows the same steps of standard HPM until (5), next we apply Laplace transform on both sides of homotopy equation (5), to obtain
8

using the differential property of L.T, we have (Spiegel 1988)
9

or
10

applying inverse Laplace transform to both sides of (10), we obtain
11

Assuming that the solutions of (3) can be expressed as a power series of *p*12

Then substituting (12) into (11), we get
13

comparing coefficients of *p*, with the same power leads to
14

Assuming that the initial approximation has the form: *U*(0) = *u*_0_ = *α*_0_, = *α*_*n* − 1_; *U*′(0) = *α*_1_,.., *U*^*n* − 1^(0) therefore the exact solution may be obtained as follows
15

## Governing equations

**Figure 1:**
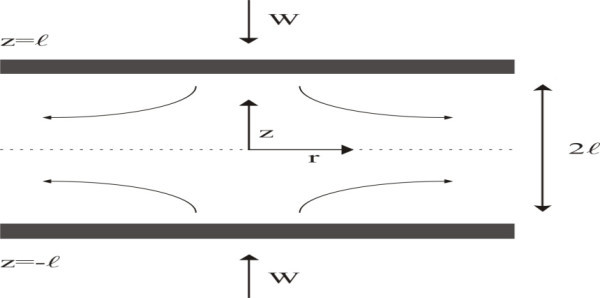
**Shows an axisymmetric fluid, squeezed between two infinite parallel plates.**

The purpose of this job is the search for an approximate solution for the nonlinear problem, which describes a viscous, incompressible fluid, squeezed between two infinite parallel plates, so that the plates are moving towards each other with a certain velocity, say *W* (see Figure 1).

The basic equations for this case, in the absence of body forces are given by
1617

where

 is the velocity vector, *ρ* the density, *D* represents the material time derivative, and *T* is the stress tensor, which is given by  where *μ* is the dynamic viscosity of the fluid and *P* the pressure.

By symmetry arguments, the problem involves a steady axisymmetric flow, so that  is represented by
18

Next, in order to simplify the analysis, we introduce the stream function *ψ*(*r*, *z*, *t*) defined by
19

Thus, we have to determine only one unknown function *ψ*(*r*, *z*, *t*), rather than the two functions *u*_*r*_(*r*, *z*, *t*) and *u*_*z*_(*r*, *z*, *t*).

It’s easy to show that the continuity equation (16) is identically satisfied using (19). Substituting (19) into the z and r components of (17) we obtain
2021

where the differential operator *E*^2^ is given by


After eliminating the pressure from the above equations, we obtain the following equation for *ψ*(*r*, *z*, *t*)
22

We will assume that W is small enough so that, during the process, the gap 2*l* between the plates changes little and it can be considered approximately constant (see Figure 1).

Under these conditions the flow can be considered quasi-steady (Hughes & Brighton 1967; Papanastasiou et al. 2000), and therefore *ψ* = *ψ*(*r*, *z*), so that (22) is rewritten as
23

with the following boundary conditions (see Figure 1)
24

Following (Stefan 1874), (23) can be expressed as a four order ordinary differential equation, by using of the substitution
25

In view of (25), (23) and (24) become
26

with the boundary conditions
27

(see for example that, after substituting (25) into the second equation of (19), we obtain *u*_*z*_(*r*, *z*) = − 2*F*(*z*), in such a way that from (24) is obtained *u*_*z*_(*r*, 0) = − 2*F*(0) = 0, and so on). In order to facilitate the evaluation of (26) we introduce the following dimensionless parameters given by
28

so that, (26) and (27) adopt the form
2930

where we have dropped * for simplicity.

## Case study

The objective of this section is employ LT-HPM, to find an analytical approximate solution for the nonlinear problem given by (29) and (30).
31

from (28), *ϵ* is a positive parameter.

It is possible to find a handy solution by applying the LT-HPM method.

Identifying terms:
3233

where prime denotes differentiation respect to z.

In order to obtain an approximate analytical solution for nonlinear problem (31), we construct a homotopy in accordance with (4)
34

or
35

Applying Laplace transform algorithm we get
36

as it is explained in (Spiegel 1988), it is possible to rewrite (36) as
37

where we have defined *Y*(*s*) = *ℑ*(*F*(*z*).

After applying the initial conditions, *F*(0) = 0, *F*″(0) = 0, the last expression can be simplified as follows
38

Solving for *Y*(*s*) and applying Laplace inverse transform *ℑ*^− 1^39

where, we have defined *A* = *F*′(0), *B* = *F*‴(0).

Next, we assume a series solution for *F*(*z*), in the form
40

and by choosing
41

as the first approximation for the solution of (31) that satisfies the conditions *F*(0) = 0, *F*″(0) = 0.

Substituting (40) and (41) into (39), we get
42

On comparing the coefficients of like powers of *P* we have
434445

Solving the above Laplace transforms for *v*_0_(*z*), *v*_1_(*z*), *v*_2_(*z*),.. we obtain
464748

and so on.

By substituting solutions (46)-(48) into (15) and calculating the limit when *p* → 1, results in a second order approximation
49

On the other hand, the derivative of (49) is given by
50

In order to calculate the values of *A* and *B*, we require that equations (49) and (50) satisfy the boundary conditions *F*(1) = 1, *F*′(1) = 0, respectively. This gives rise to a system of equations for the unknowns *A* and *B*, above mentioned. Considering as cases study *ϵ*=1 and *ϵ*=2 we obtain the values
51

and
52

respectively.

Substituting (51) into (49), we obtain
53

On the other hand, substituting (52) into (49), we obtain
54

## Discussion

**Figure 2:**
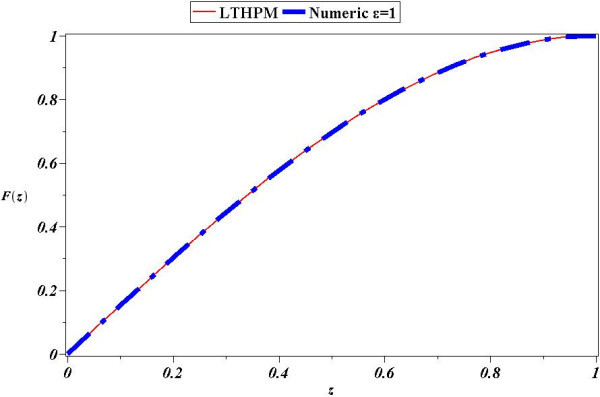
**Comparison between numerical solution of (** **31** **) for** ***ϵ*** **=1 and LT-HPM approximation (** **53** **).**

**Figure 3:**
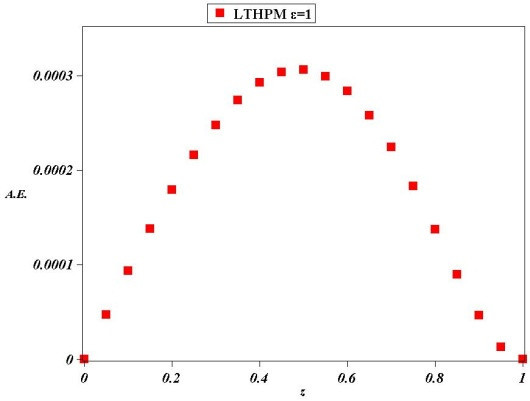
**Absolute Error (A.E.) between numerical solution of (** **31** **) for** ***ϵ*** **=1 and LT-HPM approximation (** **53** **).**

**Figure 4:**
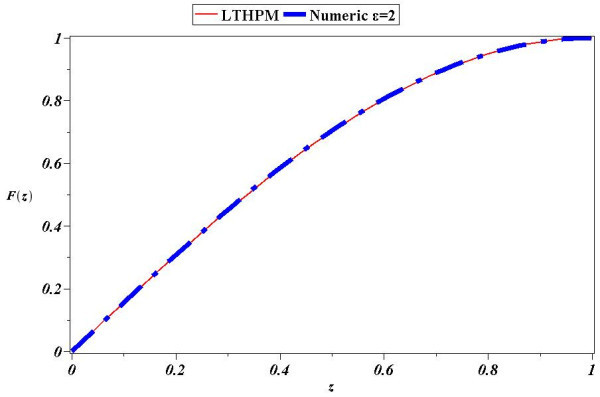
**Comparison between numerical solution of (** **31** **) for** ***ϵ*** **=2 and LT-HPM approximation (** **54** **).**

**Figure 5:**
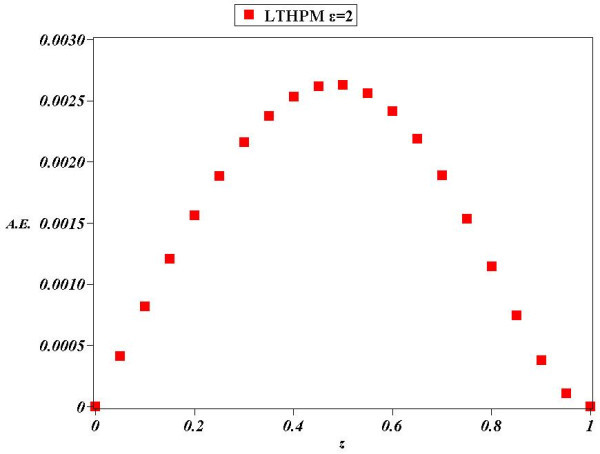
**Absolute Error (A.E.) between numerical solution of (** **31** **)** ***ϵ*** **=2 and LT-HPM approximation (** **54** **).**

In this work LT-HPM was used in the search for a handy accurate analytical approximate solution, for the nonlinear fourth order ordinary differential equation with finite boundary conditions, which describes the problem of squeezing flow between two infinite parallel plates slowly approaching each other. Figures 2, 3, 4 and 5, which compare our approximations with the numerical solution, showed good confirmation for all cases (for comparison purposes, we considered that the “exact” solution is computed using a scheme based on a trapezoid technique combined with a Richardson extrapolation as a build-in routine from Maple 17. Moreover, the routine was configured using an absolute error (A.E.) tolerance of 10^− 12^). Since LT-HPM is expressed in terms of initial conditions for a given differential equation (see (14)), our procedure was aimed to express the approximate solutions in terms of two unknown quantities *A* = *F*′(0), *B* = *F*‴(0). We noted that these values can be determined requiring that approximate solution satisfies the couple of boundary conditions *F*(1) = 1 *F*′(1) = 0, from which one obtain an algebraic system of equations for the unknowns *A* and *B* above mentioned, whose solution concludes the procedure.

Figure 2 shows the comparison between numerical solution and approximate solution (53) for *ϵ*=1. It can be noticed that curves are in good agreement, from which is clear the accuracy of our approximation, as a matter of fact Figure 3 shows that the biggest absolute error (A.E) of (53) is scarcely of 0.0003, which is remarkably precise, above all taking into account that (53) is just a second order approximate solution for (31).

Next, we found an approximate solution for the case of parameter *ϵ*=2 Figure 4 shows that (54) is an accurate analytical approximate solution for (31); from Figure 5 we deduce that the biggest absolute error (A.E) is of little more than 0.0025, whereby it is clear the reliability of LT-HPM method in the search for approximate solutions of nonlinear problems with finite boundary conditions. An important fact from LT-HPM follows from equations as (31), which can be written in the form *L*(*z*) + *ϵN*(*z*) = 0 where, *L*(*z*) is linear and *N*(*z*) nonlinear. It’s well known that classical methods of approximation as perturbation method PM (Holmes 1995; Chow 1995) provide in general, better results for small perturbation parameters *ϵ* < < 1 (for our case, the perturbation parameter would be small for small values of the distance between the plates and of the density of the fluid (see (28)). To be precise, *ϵ* can be visualized as a parameter of smallness, that measures how greater is the contribution of linear term *L*(*z*) than the one of *N*(*z*)_._ In general it is easier to find analytical approximate solutions to equations as (31) for small values of *ϵ* than for big values of the same. Figures 2, 3, 4 and 5 show a noticeable fact, that (53) and (54) provide a good approximation as solutions of (31), despite of the fact that perturbation parameters *ϵ*=1 and *ϵ*=2 cannot be considered small.

From the above, it is evident that for values of *ϵ* ≤ 2, the LT-HPM solution will describe efficiently the nonlinear problem (31). On the other hand, as we take bigger values of *ϵ* it will be necessary to consider higher order approximations of (15), in order to keep the accuracy, but possibly losing the handy character of our approximations. In any case, LT-HPM, is not a restricted method, to small parameters (Filobello-Nino et al. 2013). A reason by which LT-HPM applied to problems with boundary conditions is as efficient and converges so rapidly (Filobello-Nino et al. 2013), is that unlike other methods (for instance HPM) which include the boundary conditions from the beginning of the problem at the lowest order approximation, LT-HPM estimates one of the initial conditions unknown at first, requiring that the whole proposed solution satisfies one of the boundary conditions (the other boundary condition is satisfied from the beginning of the procedure), thus is ensured that the approximate solution fits correctly on both boundaries of the interval.

Is expected to be possible to apply other methods to solve the nonlinear problem proposed (31), for example, HPM and HAM. Since HPM is a particular case of the parameters of HAM (*h* = − 1), it is expected that in general, the approximation obtained with HAM turns out to be more accurate, because its region of convergence is based on adjusting of that parameter, while HPM corresponds to a fixed value of the aforementioned parameter and therefore is limited. However, HAM requires sometimes longer expressions, for getting accurate results, such as was reported in (Ran et al. 2009; Murad et al. 2011), where homotopy analysis method was employed to provide an approximate solution of (31). Although the solutions reported to have good accuracy, they require of major order of approximation (in (Ran et al. 2009), for example, approximations were calculated up to fiftieth order), besides generally, HAM is more complicated to applications than LT-HPM, because their approximate expressions are too long and cumbersome, in contrast to expressions like (49), (53) and (54).

**Figure 6:**
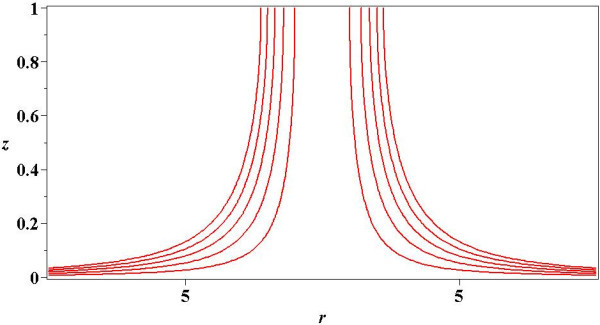
**Streamlines for** ***ϵ*** **=1 using (** **25** **) and (** **53** **).**

Simplicity of our approximations (53) and (54) allow to obtain a simple analytical expression for the velocity field, for which would be sufficient to replace them in (25) and then, the results obtained in this way, into the expressions for the components of velocity (19). Figure 6 exemplifies the case *ϵ*=1. It shows a sketch for several streamlines for various values of the distance *r*, and therefore, provides a graphical representation of the velocity field, because as it is well known, streamlines are lines in the flow field that are everywhere tangent to the velocities (Hughes & Brighton 1967).

## Conclusions

In this paper LT-HPM was employed to provide an approximate analytical solution for the fourth order nonlinear differential equation which describes a squeezing flow between two infinite plates with, mixed boundary conditions defined on a finite interval. LT-HPM method expresses the problem of finding an approximate solution for a nonlinear ordinary differential equation, in terms of solving an algebraic system of equations for some unknowns initial conditions. Figures 2, 3, 4 and 5, show the efficiency of this method in the search for solutions of nonlinear boundary value problems.

The above is an additional advantage for the method, considering that LT-HPM does not need to solve several recurrence differential equations, by which is a tool efficient, useful and precise in practical applications.
